# Local radiotherapy of exposed murine small bowel: Apoptosis and inflammation

**DOI:** 10.1186/1471-2482-8-1

**Published:** 2008-01-03

**Authors:** Andrea Polistena, Louis Banka Johnson, Salomé Ohiami-Masseron, Lena Wittgren, Sven Bäck, Charlotte Thornberg, Virgil Gadaleanu, Diya Adawi, Bengt Jeppsson

**Affiliations:** 1 Department of Surgery, Malmö University Hospital, Lund University, Malmö, Sweden; 2 Department of Radiation Physics Malmö University Hospital, Lund University, Malmö, Sweden; 3 Department of Pathology, Malmö University Hospital, Lund University, Malmö, Sweden; 4 I Scuola di Specializzazione in Chirurgia Generale, Dipartimento "Pietro Valdoni", Università degli Studi di Roma "La Sapienza", Rome, Italy

## Abstract

**Background:**

Preoperative radiotherapy of the pelvic abdomen presents with complications mostly affecting the small bowel. The aim of this study was to define the features of early radiation-induced injury on small bowel.

**Methods:**

54 mice were divided into two groups (36 irradiated and 18 sham irradiated). Animals were placed on a special frame and (in the radiated group) the exteriorized segment of ileum was subjected to a single absorbed dose of 19 or 38 Gy radiation using 6 MV high energy photons. Specimens were collected for histology, immunohistochemistry (IHC) and ELISA analysis after 2, 24 and 48 hours. Venous blood was collected for systemic leucocyte count in a Burker chamber.

**Results:**

Histology demonstrated progressive infiltration of inflammatory cells with cryptitis and increased apoptosis. MIP-2 (macrophage inflammatory protein) concentration was significantly increased in irradiated animals up to 48 hours. No significant differences were observed in IL-10 (interleukin) and TNF-α (tumour necrosis factor) levels. IHC with CD45 showed a significant increase at 2 hours of infiltrating leucocytes and lymphocytes after irradiation followed by progressive decrease with time. Caspase-3 expression increased significantly in a dose dependent trend in both irradiated groups up to 48 hours.

**Conclusion:**

Acute small bowel injury caused by local irradiation is characterised by increased apoptosis of crypt epithelial cells and by lymphocyte infiltration of the underlying tissue. The severity of histological changes tends to be dose dependent and may affect the course of tissue damage.

## Background

Radiotherapy is used in the multimodal treatment for neoplastic diseases in the pelvic abdomen [1-4]. Radiation induces injury to rapidly dividing tissues and in the pelvic region small bowel is usually affected. Acute complications consist of nausea, vomiting, abdominal pain, diarrhea, gastro-intestinal haemorrhage and bacterial infection. Chronic damage is presented as radiation enteritis, ulcerations, fibrosis, stricture formation, fistulae, malabsorption and dysmotility [5-8]. Features of radiation injury at early time points can be detected mainly by histological assesment although more sensitive evaluation can be assessed by immunohistochemistry [9].

Several animal models have been used to investigate radiation-induced damage of the small bowel. Two major murine models have been described: total body or topic abdomino-pelvic irradiation [5,10,11] and selective segmental irradiation [12,13] of an exposed portion of the small bowel. Our model is a refinement of the latter model where small bowel segments can be subjected to irradiation while minimizing secondary radiation effects to other organs, lymphoid tissue and bone marrow. Local effects of subsequent radiation injury with limited effects of immunological involvement can be more accurately assessed. The aim of this study was to define the features of early, local radiation-induced damage to small bowel, focusing on inflammatory changes and apoptosis.

## Methods

### Animals

Male C57B1/6J mice (weight 22–26 g, supplied by Taconic, Denmark) were kept under standard laboratory conditions with a 12 hour light and 12 hour dark cycle, and were allowed free access to animal food (Lactamin, Sweden) and tap water *ad libitum*. All experiments were performed in accordance with legislation on the protection of animals and were approved by the Regional Ethic's Committee for Animal Experimentation at Lund University, Sweden.

### Anesthetic and surgical procedures

The mice were anesthesized with 7.5 mg Ketamine hydrochloride (Hoffman-La Roche, Basel, Switzerland) and 2.5 mg Xylazine (Janssen Pharmaceutica, Beerse, Belgium) per 100 g body weight by intraperitoneal (*i.p*.) injection. The animals were placed in supine position on a heating pad (37°C) for maintenance of body temperature. A short midline abdominal incision (1.0–1.5 cm) was performed and a 5 cm segment of ileum located 5 cm from the ileocaecal valve was exteriorized and marked with 5-0 non-absorbable sutures. Any other visible prolapsed abdominal content was replaced back into the abdomen and the animal was placed on a specially designed frame [14] with the loop of intestine fixed between two perspex sheets. After irradiation the intestine was replaced in the abdomen and the incision closed with a polypropylene suture. To reduce water losses exposed bowels were covered with moist swabs, before and after radiotherapy.

### Irradiation protocol

54 animals were divided in two main groups: radiated mice (n = 36) and sham radiated (n = 18). All animals underwent laparotomy for exposure of the intestinal segment that was analysed. The exposed ileum was subjected to a single high absorbed dose radiation of either 19 or 38 Gy and the mouse protected by an 8 cm thick lead metal shield. The irradiations were undertaken using a clinical linear accelerator (Varian Clinac 2100C) and in normal room temperature. The method has been described elsewhere [14]. Briefly, the exteriorized mouse intestine was positioned between 1.5 cm thick perspex slabs (10 × 15 cm^2^) to sufficiently reduce secondary radiation scatter and thereby accomplish a reproducible and homogenous dose distribution. The (adjustable) spacing between the slabs was just sufficient to support the exteriorized mouse intestine (maximum 0.5 cm). The absorbed dose was verified with independent measurements and was found to be within 5% throughout the intended volume using this technique. The use of an asymmetrically half blocked 6 MV beam and extra lead shielding assured that the treatment field perfectly fitted the exteriorized intestine while the remaining mouse body was kept outside the radiation beam. The absorbed dose rate was 3.2 Gy/minute and consequently the irradiation time for each animal was approximately 6 minutes for 19 Gy (n = 18) and 12 minutes for 38 Gy (n = 18). An absorbed dose of 19 Gy delivered to the intestine is known to cause consistent structural, cellular, and molecular changes [15]. The sham operated mice underwent the same surgical procedure except irradiation. Exposure time from surgery, through irradiation to wound closure was kept at a minimum in order to reduce stress and trauma levels, the whole procedure taking approximately 15 minutes.

### Collection of samples

Specimens were collected for the 3 different time points (2, 24 and 48 hours). At each timepoint 3 groups (19 Gy, 38 Gy and Sham) consisting of 6 animals per group were studied. A second laparotomy was performed; the marked bowel segment was exteriorized and excised. It was then divided into segments of 0.5–1.0 cm lengths which were weighed and stored for histology, immunohistochemistry and cytokine analysis.

### Histological study

Samples were collected at different time points after irradiation. The marked irradiated bowel segment was excised and rinsed with normal saline. The samples were fixed in 4% buffered paraformaldehyde, dehydrated in graded ethanol and embedded in paraffin. The sections were cut on a microtome and mounted on glass slides. The slides were stained with hematoxylin and eosin for histological evaluation under light microscopy, which was done by the pathologist in a blinded fashion.

### Cytokines

Tissue samples were washed in PBS containing penicillin, streptomicin and fungizon (100 U/ml) and kept cool in cold serum-free medium (DMEM). Bowel was incubated in 1 ml of DMEM solution 10% FCS and penicillin, streptomycin for 24 hours (37°C) in a 12-wells plate [16]. The cultured medium was harvested and stored in -20°C until analysis of MIP-2 (Macrophage inflammatory protein-2), TNF-α (Tumour necrosis factor-α) and IL-10 (Interleukin-10) using a colorimetric sandwich ELISA kit with recombinant murine proteins as standard (Quantikine, R&D Systems, Europe). The minimal detectable protein concentration in these kits is < 1.5 pg/ml for MIP-2, <5.1 pg/ml for TNF-α and < 4.0 pg/ml for IL-10.

### Immunohistochemistry

For immunohistochemistry, standard avidin-biotin procedures for mouse CD45 (defined as leucocyte common antigen) [17-20] and for active caspase-3 were used. After deparaffinization and washing in phosphate buffered solution (PBS), endogenous peroxidase activity was blocked by incubating the sliced sections in 3% hydrogen peroxide in PBS for 10 minutes. For CD45 analysis the slides were fixed with BD Retrievagen fixative A (BD Biosciences, Europe) and then incubated overnight at 60°C. Slides for Caspase-3 were microwave-treated [21]. The sections were blocked in 5% fetal calf serum with PBS for 30 minutes and then incubated for 1 hour at room temperature with 1:100 anti mouse CD45 monoclonal antibody (RnD Systems Inc., Minneapolis, USA, cat n° MAB114) and with 1:750 anti-active human-mouse caspase-3 affinity-purified rabbit antibody (RnD Systems Inc., Minneapolis, USA, cat n° AF835) respectively. Biotin-conjugated secondary antibody and streptavidin-conjugated horseradish peroxidase (DAKO, USA) were applied to sections for 45 minutes at room temperature, and developed using the 3,3'-diaminobenzidine (DAB) as substrate. Finally counterstaining with haematoxylin for both CD45 and caspase was performed. Sections were mounted and leucocyte and apoptotic cell counts were determined on randomly selected areas using the point counting technique by a blinded observer. Each slide contains 3 sections and from each section we choose randomly 3 areas under light microscopy with a high power field ×100. We then take the mean value of all the counts in each slide.

### Statistical analysis

Sham and radiated groups were statistically analysed by one-way analysis of variance (ANOVA) with a post hoc Turkey test for all pairwise multiple comparison procedures. Non-parametrically distributed groups were studied by the Kruskal-Wallis analysis of variance on ranks. A probability (P) value ≤ 0.05 was accepted as significant. Differences were expressed as mean values ± SEM.

## Results

All animals survived the operation and radiotherapy sessions

### Histology

**Figure 1:**
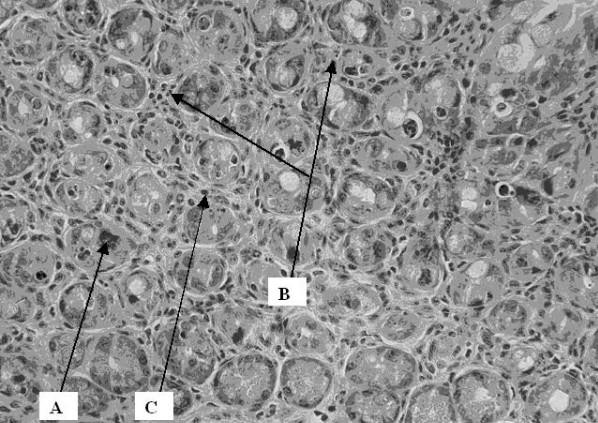
Cross section of intestinal wall 48 hrs after 19 Gy irradiation. An increase in apoptosis (A), intraepithelial granulocytes (B) and lymphocytes (C) was observed. The slides were stained with hematoxylin and eosin for histological evaluation under light microscopy, which was done by the pathologist in a blinded fashion.

**Figure 2:**
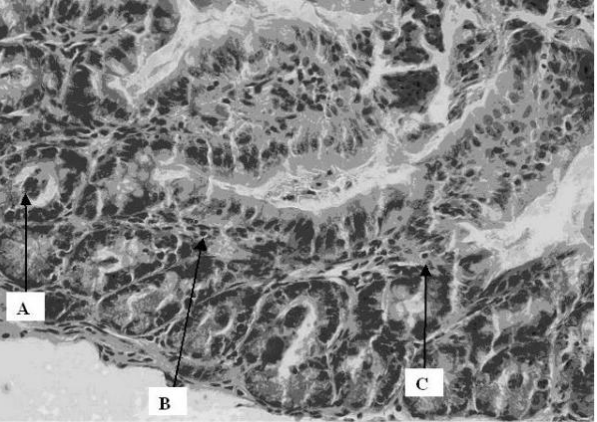
Cross section of intestinal wall 48 hrs after 38 Gy irradiation. An increase in apoptosis (A), intraepithelial granulocytes (B) and lymphocytes (C) was observed with degenerative epithelium and granulocyte exudate in the lumen (between the villi and even in the crypts, suggesting cryptitis). The slides were stained with hematoxylin and eosin for histological evaluation under light microscopy, which was done by the pathologist in a blinded fashion.

At the normal controls: No inflammatory changes were observed 2 hours after surgery. There was similarly no major difference at 24 and 48 hours except for the presence of some granulocytes and a few apoptotic cells in the crypts. At 19 Gy: normal cell structure was kept at 2 hours, apart from some dilated peripheral vessels, indicating hyperaemia. At 24 hours some lymphocytes, dilated vessels and some apoptotic cells were present in the crypts. At 48 hours, signs of hyperaemia, a further increase in apoptosis and mitosis (active cells) as well as an increase in granulocytes in the epithelium (Figure 1), lamina propria and subserosa was observed. At 38 Gy: an increase in the inflammatory infiltrate was observed. At 2 hours some inflammatory cells (granulocytes) and few apoptotic cells together with other degerative epithelial cells were observed. At 24 hours we found an increase in granulocytes, massive apoptosis and increased exudative inflammation in the lumen (between the villi and even in the crypts, suggesting cryptitis). The changes after 48 hrs were similar to those found at 24 hours but to a much lesser degree (Figure 2).

### Caspase-3 Immunohistochemistry staining

**Figure 3:**
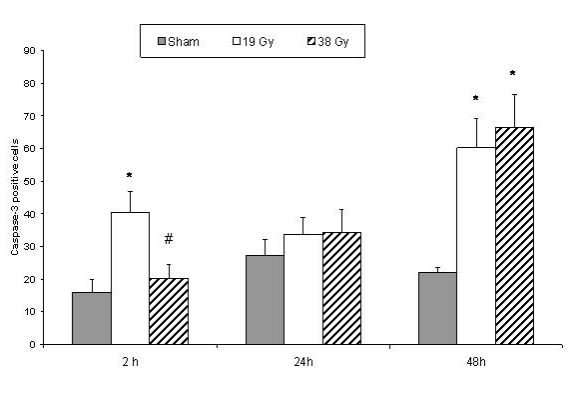
Caspase-3 positive stained cells in small bowel tissue comparing sham and different doses of radiation within each time point. * denotes p < 0.05 compared to sham group, # denotes p < 0.05 compared to 19 Gy group.

Two hours after radiation there was a significant increase in caspase-3 positive cells in the 19 Gy group compared to sham and 38 Gy irradiated groups (Figure 3). 24 Hours after radiation there was no difference between the groups (Figure 3). At 48 hours there was a significant increase in both irradiated groups compared to the sham irradiated group (Figure 3).

### CD 45 Immunohistochemistry staining

**Figure 4:**
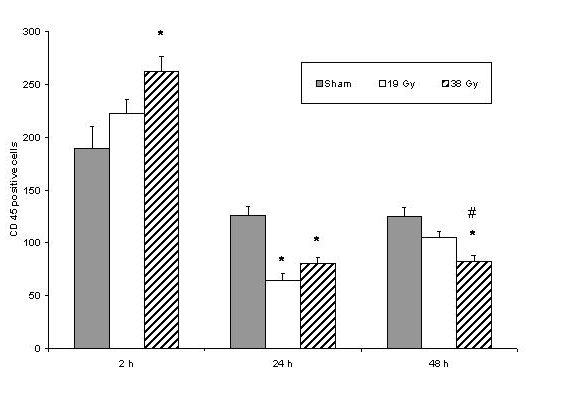
CD45 positive stained cells in small bowel tissue comparing sham and different doses of radiation within each time point. * denotes p < 0.05 compared to sham group, # denotes p < 0.05 compared to 19 Gy group.

Two hours after irradiation there was an increase in CD45 positive cells in the irradiated group exposed to 38 Gy compared to the sham irradiated group (Figure 4). 24 hours after irradiation there was a significant decrease in both irradiated groups compared to sham irradiation (Figure 4). 48 hours after irradiation, there was a decrease in both irradiated groups with a significant difference in the 38 Gy group compared to both sham and 19 Gy irradiated groups.

### Cytokines

**Figure 5:**
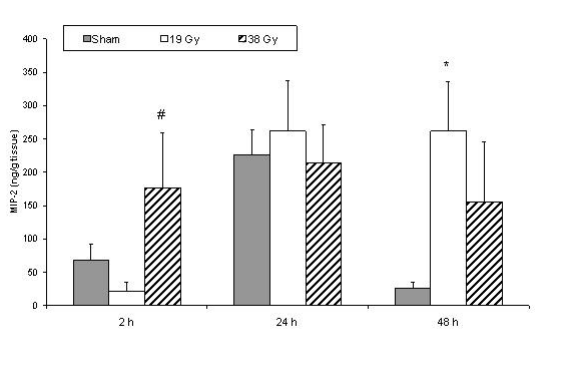
MIP-2 concentration in small bowel tissue comparing sham and different doses of radiation within each time point. * denotes p < 0.05 compared to sham group, # denotes p < 0.05 compared to 19 Gy group.

Two hours after irradiation there was a significant increase in MIP-2 in 38 Gy group compared to 19 Gy group (Figure 5). 24 hours after irradiation there was no difference between the groups. 48 hours after irradiation, there was an increase in both irradiated groups with a significant difference in the 19 Gy group compared to sham irradiated group. When comparing the different time points in each treatment group, we found difference in the sham and 19 Gy groups while there was no difference within the 38 Gy groups. MIP-2 activity showed the significantly highest values in the sham group at 24 hours followed by a significant decrease after 48 hours. In the 19 Gy groups higher values were observed at 24 and 48 hours compared to 2 hours values. TNF-α and IL-10 levels did not exhibit any significant changes in neither sham nor irradiated groups.

## Discussion

The local effect of radiotherapy on an exposed segment of small bowel includes early increases in MIP-2 levels, a decrease in CD 45 positive cells over time and increased dose dependent apoptosis. Many studies show a general over-expression of pro-inflammatory proteins such as MIP-2 and TNF-α after whole body rodent irradiation [22,23]. Macrophage inflammatory protein-2 (MIP-2) is a CXC chemokine and its secretion enhanced by inflammatory stimuli. There is evidence that MIP-2 is one of the major chemokines that lead to neutrophil recruitment and infiltration in several animal models of inflammation [24,25]. Some of the main pathways include the ingestion of apoptotic cells by macrophages which lead to the production of MIP-2 which is a potent chemoattractants for neutrophils. Recent reports indicate that MIP-2 is also regulated by oxygen radicals [21]. In our model there was a successive elevation of all MIP-2 levels up to 24 hours after which the irradiated groups generally maintained their levels whilst the sham levels dropped at 48 hours. 19 Gy irradiation being significantly raised compared to sham treatment at this time point.

A major problem in planning radiotherapy is acheiving an optimal radiation schedule, balancing dose, fractionation and exposure time for maximum tumoricidal effect. At the same time there must be protection against injury to healthy surrounding tissues. In radiotherapy of the abdominopelvic region the small bowel is frequently injured and the severity of mucosal damage depends on the balance between epithelial denudation and proliferation. When the rate at which surviving epithelial cells killed exceeds the maximum rate at which new cells are replaced the injury is more evident. The deeper the lesion is the slower the re-epithelization with loss of barrier function since the main damage is to the epithelial stem cells at the basement membrane zone.

An increased leucocyte intravascular rolling and adhesion after radiation with resultant inflammatory infiltrate, composed mainly of lymphocytes has been observed in the lamina propria of intestinal villi [9]. The CD45 antigen family is a group of high molecular weight glycoproteins expressed on the membranes of all leucocytes (haematopoetic cells). Its role in membrane signal transduction and lymphocyte activation is well defined and allows a specific function as marker of B and T cells. Our CD 45 values show a sharp increase of in all the groups at 2 hours but with the 38 Gy group significantly increased compared to sham. The values then fell again at 24 hours, with a reduction of the irradiated groups to less than half of their values at 2 hours. All levels continued to be maintained in generally the same way from 24 to 48 hours post-irradiation. Radiation thus tends to reduce the number of active CD45 bearing leucocytes/lymphocytes locally in the inflammatory infiltrate of the irradiated tissue. The total leucocyte count in this study did not show any significant changes in the different groups. This may be explained by the fact that radiation was locally administered without bone marrow or systemic involvement, leaving systemic leucocyte production mainly unaffected.

In the acute phase, increase in radiation dosage leads to increase in apoptosis and granulocyte exudative inflammation mostly seen in the crypts. Some of the changes observed could most possibly be the pre-stadium to crypt abscess formation. Active caspase-3 immunostaining is specific for the late phase of the apoptotic process. Immunostaining technique in an in vivo irradiated animal model showed a significant expression of caspase-3 activity in γ-radiated mice at the crypt level although no difference was noticed at the villus tip [26]. This phenomenon is clearly observed in our experiment and is explained by the fact that proliferating crypt cells are more susceptible to radiation-induced apoptosis as opposed to already differentiated villi epithelial cells [27]. Our experimental results showed caspase expression from the 2 hour time point increasing to maximum expression at 48 hours after both 19 and 38 Gy irradiation. A significant expression compared to sham at this time point. This trend corresponds to results of earlier in vivo studies [28].

A high level of TNF-α is often found after general inflammatory stimulus and radiation and it has been shown to be one of the most important inducers of the apoptotic process [27]. Nevertheless in our setting no detectable level of TNF-α could be measured, suggesting that either the local effect of radiotherapy was not enough to affect a noticable stimulus for TNF-α production or that probably wrong timing and/or incorporation of a less sensitive method of analysis could be an explanation of this finding. No significant difference was either observed in IL-10 expression within our groups.

## Conclusion

Early local radiation-induced tissue injury of small bowel in this refined rodent model shows a progressive, infiltration of inflammatory cells into tissues probably mediated by the release of MIP-2 and a dose related increase of apoptosis. High irradiation may lead to a shift from crypt apoptosis to cryptitis (the advanced forms being most probably a pre-stage to crypt abscess formation) and inevitably necrosis. This model has proven to be good at studying the early local effects of radiotherapy on the bowel and has the potential of even being a useful model for long term effect study.

## Competing interests

The author(s) declare that they have no competing interests.

## Authors' contributions

AP: Participated in the design of the study and performed experimental studies and drafted the manuscript.

LBJ: Designed the study and participated in construction of the chamber. Performed experimental studies and drafted the manuscript.

SOM: Performed experimental studies.

LW: Participated in the radiological design of the study, construction of the chamber and the implementation of radiotherapy.

SB: Participated in the radiological design of the study, chamber and the implementation of radiotherapy.

CT: Participated in the implementation of radiotherapy.

VC: Performed the histological analysis.

DA: Participated in the design of the study, performance of experimental studies, drafting the manuscript.

BJ: Conceived the design and participated in construction of the chamber. Co-ordination of the study as well as supervision and draft of the manuscript.

All authors have read and approved the final version of the manuscript.

## Pre-publication history

The pre-publication history for this paper can be accessed here:



## References

[B1] Ahmadu-Suka F, Gillette EL, Withrow SJ, Husted PW, Nelson AW, Whiteman CE (1988). Pathological response of the pancreas and duodenum to experimental intraoperative irradiation. Int J Oncol Biol Phys.

[B2] Avizonis VN, Sause WT, Noyes RD (1989). Morbidity and mortality associated with intraoperative radiotherapy. J Surg Oncol.

[B3] Dubois JB (1997). Effects tardifs de la radiotherapie peroperatoire. Cancer/Radiother.

[B4] Eble MJ, Lehnert T, Treiber M, Latz D, Herfarth C, Wannenmacher M (1998). Moderate dose intraoperative and external beam radiotherapy for locally recurrent rectal carcinoma. Radiother Oncol.

[B5] Freeman SL, Hossain M, Mac Naughton WK (2001). Radiation-induced acute intestinal inflammation differs following total-body versus abdominopelvic irradiation in the ferret. Int J Radiat Biol.

[B6] Hauer-Jensen M (1990). Late radiation injury of the small intestine Clinical pathophysiological and radiobiologic aspects. A review. Acta Oncol.

[B7] Mandel L, Svoboda V (1991). The gastrointestinal post-irradiation syndrome. SB ved PR lek FAK karlovy University Hradci Kralove Suppl.

[B8] Sindelar WF, Tepper JE, Kinsella TJ, Barnes M, DeLuca AM, Terrill R, Matthews D, Anderson WJ, Bollinger BK, Johnstone PA (1994). Late effects of intraoperative radiation therapy on retroperitoneal tissues, intestine and bile duct in a large animal model. Int J Radiat Oncol Biol Phys.

[B9] Richter KK, Langberg CW, Sung CC, Hauer-Jensen M (1997). Increased transforming growth factor beta (TGF-beta) immunoreactivity is independently associated with chronic injury in both consequential and primary radiation enteropathy. Int J Radiat Oncol Biol Phys.

[B10] Mollà M, Gironella M, Salas A, Miquel R, Pérez-del-Pulgar S, Conill C, Engel P, Biete A, Piqué JM, Panés J (2001). Role of P-selectin in radiation-induced intestinal inflammatory damage. Int J Cancer.

[B11] Payne CM, Bjore CG, Schultz DA (1992). Change in the frequency of apoptosis after low- and high-dose X-irradiation of human lymphocytes. J Leukoc Biol.

[B12] Delaney JP, Bonsack ME, Felemovicius I (1994). Radioprotection of the rat small intestine with topical WR-2721. Cancer.

[B13] Zheng H, Wang J, Koteliansky VE, Gotwals PJ, Hauer-Jensen M (2000). Recombinant soluble transforming growth factor beta type II receptor ameliorates radiation enteropathy in mice. Gastroenterology.

[B14] Johnson LB, Riaz AA, Adawi D, Wittgren L, Bäck S, Thornberg C, Osman N, Gadaleanu V, Thorlacius H, Jeppsson B (2004). Radiation enteropathy and leucocyte-endothelial cell reactions in a refined small bowel model. BMC Surgery.

[B15] Bjelkengren G, Aronsen KF, Augustsson NE, Borgstrom S, Lindstrom C, Nylander G (1995). Radioprotective effect of local administration of lysine-vasopressin and triglycyl-lysine-vasopressin on the rectal mucosa in rats. Acta Oncol.

[B16] Riaz AA, Schramm R, Sato T, Menger MD, Jeppsson B, Thorlacius H (2003). Oxygen radical-dependent expression of CXC chemokines regulate ischemia/reperfusion-induced leukocyte adhesion in the mouse colon. Free Radic Biol Med.

[B17] Caballero T, Nogueras F, Medina MT, Caracuel MD, de Sola C, Martínez-Salmerón FJ, Rodrigo M, García del Moral R (1995). Intraepithelial and lamina propria leucocyte subsets in inflammatory bowel disease: an immunohistochemical study of colon and rectal biopsy specimens. J Clin Pathol.

[B18] Hase T, Shinta K, Murase T, Tokimitsu I, Hattori M, Takimoto R, Tsuboi R, Ogawa H (2000). Histological increase in inflammatory infiltrate in sun-exposed skin of female subjects: the possible involvement of matrix metalloproteinase-1 produced by inflammatory infiltrate on collagen degradation. Br J Dermatol.

[B19] Simon DI, Chen Z, Seifert P, Edelman ER, Ballantyne CM, Rogers C (2000). Decreased neointimal formation in Mac-1-/- mice reveals a role for inflammation in vascular repair after angioplasty. J Clin Invest.

[B20] Uzawa A, Suzuki G, Nakata Y, Akashi M, Ohyama H, Akanuma A (1994). Radiosensitivity of CD45R0+ memory and CD45R0- naive T cells in culture. Radiat Res.

[B21] Nossuli TO, Frangogiannis NG, Knuefermann P, Lakshminarayanan V, Dewald O, Evans AJ, Peschon J, Mann DL, Michael LH, Entman ML (2001). Brief murine myocardial I/R induces chemokines in a TNF-alpha-independent manner: role of oxygen radicals. Am J Physiol Heart Circ Physiol.

[B22] Kyrkanides S, Moore AH, Olschowka JA, Daeschner JC, Williams JP, Hansen JT, Kerry O'Banion M (2002). Cyclooxygenase-2 modulates brain inflammation-related gene expression in central nervous system radiation injury. Molec Brain Res.

[B23] Weichselbaum RR, Kufe DW, Hellman S, Rasmussen HS, King CR, Fischer PH, Mauceri HJ (2002). Radiation-induced tumor necrosis factor-α expression: clinical application of transcriptional and physical targeting of gene therapy. Lancet Oncol.

[B24] Han XB, Liu X, Hsueh W, De plaen IG (2004). Macrophage inflammatory protein-2 mediates the bowel injury induced by platelet-activating factor. Am J Physiol Gastrointest Liver Physiol.

[B25] Uchimura E, Watanabe N, Niwa O, Muto M, Kobayashi Y (2000). Transient infiltration of neutrophils into the thymus in association with apoptosis induced by whole-body X-irradiation. J Leukoc Biol.

[B26] Marshman E, Ottewell PD, Potten CS, Watson AJM (2001). Caspase activation during spontaneous and radiation-induced apoptosis in the murine intestine. J Pathol.

[B27] Ramachandran A, Madesh M, Balasubramanian KA (2000). Apoptosis in the intestinal epithelium: Its relevance in normal pathophysiological conditions. J of Gastr Hepat.

[B28] Labejof LP, Galle P, Mangabeira PA, de Oliveira AH, Severo MI (2002). Histological changes in rat duodenum mucosa after whole-body gamma irradiation. Cell Mol Biol.

